# Age-Associated Disruption of Molecular Clock Expression in Skeletal Muscle of the Spontaneously Hypertensive Rat

**DOI:** 10.1371/journal.pone.0027168

**Published:** 2011-11-04

**Authors:** Mitsunori Miyazaki, Elizabeth Schroder, Stephanie E. Edelmann, Michael E. Hughes, Karl Kornacker, C. William Balke, Karyn A. Esser

**Affiliations:** 1 Department of Physiology, Center for Muscle Biology, University of Kentucky, Lexington, Kentucky, United States of America; 2 Department of Cellular and Molecular Physiology, Yale School of Medicine, New Haven, Connecticut, United States of America; 3 Division of Sensory Biophysics, Ohio State University, Columbus, Ohio, United States of America; 4 Clinical and Translational Science Institute and the Department of Medicine, University of California San Francisco, San Francisco, California, United States of America; Vanderbilt University, United States of America

## Abstract

It is well known that spontaneously hypertensive rats (SHR) develop muscle pathologies with hypertension and heart failure, though the mechanism remains poorly understood. Woon et al. (2007) linked the circadian clock gene *Bmal1* to hypertension and metabolic dysfunction in the SHR. Building on these findings, we compared the expression pattern of several core-clock genes in the gastrocnemius muscle of aged SHR (80 weeks; overt heart failure) compared to aged-matched control WKY strain. Heart failure was associated with marked effects on the expression of *Bmal1*, *Clock* and *Rora* in addition to several non-circadian genes important in regulating skeletal muscle phenotype including *Mck*, *Ttn* and *Mef2c*. We next performed circadian time-course collections at a young age (8 weeks; pre-hypertensive) and adult age (22 weeks; hypertensive) to determine if clock gene expression was disrupted in gastrocnemius, heart and liver tissues prior to or after the rats became hypertensive. We found that hypertensive/hypertrophic SHR showed a dampening of peak *Bmal1* and *Rev-erb* expression in the liver, and the clock-controlled gene *Pgc1α* in the gastrocnemius. In addition, the core-clock gene *Clock* and the muscle-specific, clock-controlled gene *Myod1*, no longer maintained a circadian pattern of expression in gastrocnemius from the hypertensive SHR. These findings provide a framework to suggest a mechanism whereby chronic heart failure leads to skeletal muscle pathologies; prolonged dysregulation of the molecular clock in skeletal muscle results in altered *Clock*, *Pgc1α* and *Myod1* expression which in turn leads to the mis-regulation of target genes important for mechanical and metabolic function of skeletal muscle.

## Introduction

The role of the molecular clock as an underlying factor contributing to cardiovascular and skeletal muscle disease is a new but growing area of research. It is now recognized that most, if not all, cells in the body contain a self-sustaining molecular circadian clock [1]. In general, the synchronization of all the body's clocks is orchestrated by a central clock (SCN: suprachiasmatic nucleus) located in the hypothalamus acting through neurohumoral mechanisms [2]. The synchronization of circadian clocks has been experimentally shown to provide an adaptive advantage by enhancing an organisms ability to respond to daily changes in light, temperature and humidity [3]. Pathologies emerge when there is a misalignment between internal circadian rhythms and daily cycles in environmental cues such as light which can occur when the expression of the molecular clock becomes shifted or dampened [4].

Circadian rhythms, endogenously generated rhythms with ≈24 hour-period, are driven by an intrinsic molecular clock which works as a transcription/translation feedback system. In mammals, the proteins encoded by core-clock genes, *Bmal1* and *Clock*, dimerize to drive transcription of *Period* (*Per*) and *Cryptochrome* (*Cry*) and the protein products of these genes down-regulate BMAL1 and CLOCK function. BMAL1∶CLOCK heterodimers directly regulate the expression of a group of genes referred to as primary clock-controlled genes (CCGs). Many other genes are expressed in a circadian manner and these genes are indirectly influenced by the core clock factors as well as environmental cues, such as hormones like melatonin and cortisol [5]–[13].

Woon and colleagues (2007) identified polymorphisms in the promoter region of *Bmal1* (Brain and muscle Arnt-like protein-1) that were within the same congenic interval associated with hypertension in the spontaneously hypertensive rat (SHR) [14]. Furthermore, the same group reported a significant genetic association of *Bmal1* polymorphisms and hypertension and type II diabetes in humans [14]. These findings implicate the regulation of *Bmal1* expression and proper molecular clock function in the development of cardiometabolic pathologies. Consistent with these findings, Andrews and coworkers (2010) identified a common skeletal muscle pathology in two clock-compromised mouse strains that were characterized by diminished force capacity and reduced mitochondrial content and function [5].

It is well recognized that chronic heart failure (CHF) and cardiovascular disease are often associated with distinct skeletal muscle pathologies [15], [16]. This association has been shown in different rodent models of cardiovascular disease as well as in humans. These studies have reported the presence of a number of pathologies in skeletal muscle related to both function and metabolism. Specifically, structural and biochemical alterations have been demonstrated in patients with CHF, including fiber atrophy [17], fiber type transformation [18] reduced sensitivity to insulin, decrease in oxidative capacity [19] and abnormalities in mitochondrial structure [20]–[22]. The mechanism(s) underlying the development of these skeletal muscle pathologies associated with cardiovascular disease remain undefined.

The purpose of this study was to determine if changes occurred in the expression of core clock factors and CCGs in peripheral tissues either prior to or following the development of cardiovascular disease and insulin resistance. Our working hypothesis was that clock gene expression would change after the development of hypertension in muscle and non-muscle tissues. In this study we found that circadian gene expression was altered across all tissues studied (gastrocnemius, liver, heart) in the young pre-hypertensive SHR. Analysis of gene expression at the adult hypertensive/hypertrophic stage (22 week old), found that the disruption/dysregulation of clock gene expression persisted primarily in skeletal muscle of SHR compared to WKY rats. In addition, there were changes in expression of the core clock gene *Clock* and two clock-controlled genes in skeletal muscle, *Myod1* and *Pgc1α* of adult SHR. Further examination revealed, that the expression of molecular clock factors were significantly altered in the skeletal muscle of SHR at end stage heart failure. Collectively, these results suggest that genetic difference(s) in the SHR vs. WKY are associated with altered expression of the core clock factors and downstream clock controlled genes in peripheral tissues prior to the development of hypertension. We also propose that prolonged alterations of the molecular clock factors in skeletal muscle may lead to a loss of regulation of downstream targets, *Myod1* and *Pgc1α* with subsequent effects on skeletal muscle functional and metabolic pathologies described in the SHR.

## Results

### Dysregulation of core-clock genes is evident in the gastrocnemius of 8 week old SHR rats

**Figure 1:**
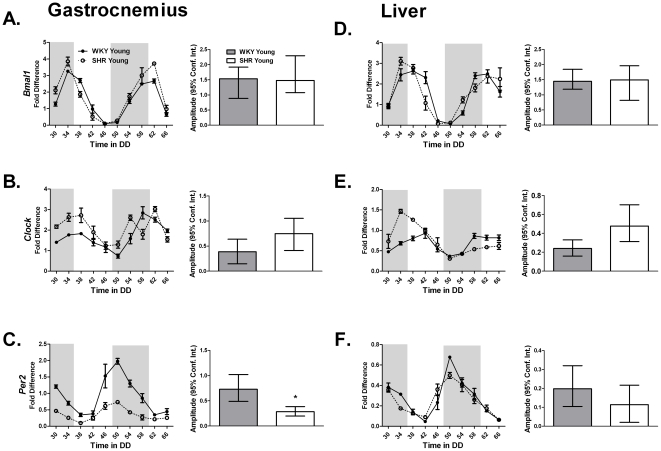
The expression of (A,D) *Bmal1*, (B,E) *Clock*, and (C,F) *Per2* from the gastrocnemius and the liver of young WKY and SHR was determined by quantitative PCR. Samples were collected every 4 hours for 40 hours. Collections were performed under red light. The dark and light bars on the graph represent extrapolated subjective day and night as defined by ZT according to the prior L∶D cycle before release into DD. A plot of amplitude with 95% confidence intervals as error bars is include next to each circadian gene expression plot.

Preliminary experiments in 80 week old, overt heart failure rats demonstrated a diminished expression of several core clock genes as well as several non-circadian genes important in regulating skeletal muscle phenotype and function (**Figures S1 and S2**) in the gastrocnemius of SHR compared to age-matched WKY rats. While it was impossible to perform a circadian time course collection on the heart failure rats because of high mortality, we were able to perform a time course on 8 week old and 22 week old SHR to determine if the onset of hypertension was associated with disruption of the circadian clock. Tissues were collected from rats every 4 hours for 40 hours. **Table 1** provides the results of the statistical analysis with the circadian parameters of the mRNA data for the core-clock genes *Bmal1*, *Clock* and *Per2* in the gastrocnemius and liver of the young WKY and SHR rats using JTK_CYCLE [23]. The core molecular clock factors, *Bmal1*, *Clock* and *Per2* exhibited circadian expression in both gastrocnemius and liver tissues (**Figure 1A–F**). Peak expression of *Per2* was reduced in the gastrocnemius (**Figure 1C**) of the young SHR. Peak *Per2* expression was unaffected in the liver and (**Figure 1F**) heart (**Figure S3C**) of the young SHR.

**Table 1:** The circadian parameters of the core-clock genes *Bmal1*, *Clock*, and *Per2* in the gastrocnemius and liver of young WKY and SHR calculated using JTK_CYCLE analysis.

Circadian Statistics Core Clock Genes				
Strain	Gene	JTK _CYCLE p value	Circadian JTK_CYCLE	JTK_CYCLE Amplitude
**Gastrocnemius**				
WKY Young	*Bmal1*	1.21E-11	Yes	1.54
SHR Young	*Bmal1*	5.73E-08	Yes	1.48
WKY Young	*Clock*	1.29E-05	Yes	0.39
SHR Young	*Clock*	3.74E-04	Yes	0.75
WKY Young	*Per2*	3.13E-09	Yes	0.73
SHR Young	*Per2*	6.20E-09	Yes	0.28
**Liver**				
WKY Young	*Bmal1*	2.20E-09	Yes	1.45
SHR Young	*Bmal1*	2.26E-06	Yes	1.49
WKY Young	*Clock*	2.43E-07	Yes	0.24
SHR Young	*Clock*	5.95E-03	Yes	0.48
WKY Young	*Per2*	8.65E-09	Yes	0.2
SHR Young	*Per2*	2.00E-05	Yes	0.11

**Figure 2:**
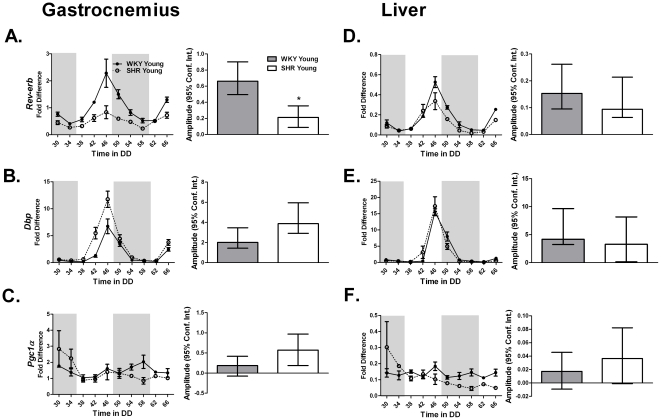
*Rev-erbα* is a core clock gene and *Dbp*, and *Pgc1α* are clock-controlled genes. The expression of (A,D) *Rev-erb*, (B,E) *Dbp*, and (C,F) *Pgc1α* in the gastrocnemius and liver of young WKY and SHR was measured by quantitative PCR. Samples were collected every 4 hours for 40 hours. Collections were performed under red light. The dark and light bars on the graph represent extrapolated subjective day and night as defined by ZT according to the prior L∶D cycle before release into DD. A plot of amplitude with 95% confidence intervals as error bars is include next to each circadian gene expression plot.

The core-clock genes *Bmal1* and *Clock* heterodimerize and transcriptionally regulate a group of direct clock-controlled genes which are thought to be necessary for the maintenance of normal cell physiology. *Rora* and *Rev-erbα* are also considered components of the molecular clock. We examined the expression patterns of the *Rev-erbα* and downstream clock-controlled genes *Dbp* and *Pgc1α*. *Rev-erbα* expression was diminished in the heart and gastrocnemius with the most dramatic effect observed in the gastrocnemius (**Figure 2A and D** **; Figure S4A**). We found that *Pgc1α* did not cycle in either the gastrocnemius or the liver at this age (**Table 2** **,** **Figure 2C and F**).

**Table 2:** The circadian parameters of the core-clock gene *Rev-erb* and the clock-controlled genes *Dbp* and *Pgc1α* in the gastrocnemius and liver of young WKY and SHR calculated using JTK_CYCLE analysis.

Circadian Statistics				
Strain	Gene	JTK _CYCLE p value	Circadian JTK_CYCLE	JTK_CYCLE Amplitude
**Gastrocnemius**				
WKY Young	*Rev erb*	3.74E-11	Yes	0.66
SHR Young	*Rev erb*	1.55E-06	Yes	0.21
WKY Young	*Dbp*	2.28E-08	Yes	2.01
SHR Young	*Dbp*	2.70E-08	Yes	3.88
WKY Young	*Pgc1α*	7.40E-02	No	0.19
SHR Young	*Pgc1α*	1.24E-01	No	0.57
**Liver**				
WKY Young	*Rev erb*	3.74E-11	Yes	0.15
SHR Young	*Rev erb*	1.04E-07	Yes	0.09
WKY Young	*Dbp*	1.04E-07	Yes	4.19
SHR Young	*Dbp*	5.19E-06	Yes	3.28
WKY Young	*Pgc1α*	1	No	0.02
SHR Young	*Pgc1α*	1	No	0.04

### Dysregulation of the molecular clock is increased in the 22 week old SHR

**Figure 3:**
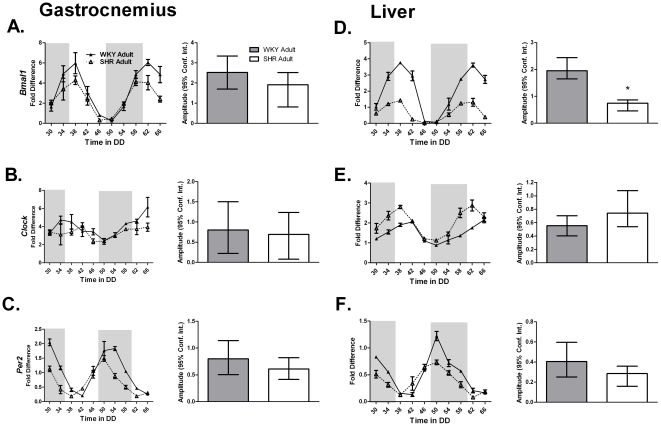
The expression of (A,D) *Bmal1*, (B,E) *Clock*, and (C,F) *Per2* from the gastrocnemius and the liver of adult WKY and SHR was determined by quantitative PCR. Samples were collected every 4 hours for 40 hours. Collections were performed under red light. The dark and light bars on the graph represent extrapolated subjective day and night as defined by ZT according to the prior L∶D cycle before release into DD. A plot of amplitude with 95% confidence intervals as error bars is include next to each circadian gene expression plot.

A circadian time-course collection was then performed with adult (22 week old) SHR and WKY rats to ascertain the expression of molecular clock factors associated with the progression of hypertension and insulin resistance. As seen with the young animals, JTK_CYCLE analysis confirmed core clock gene cycling in the gastrocnemius, liver, and heart of the WKY rats (**Table 3** **; Table S1**). We found that *Bmal1* expression was damped in the liver of the adult animals (**Figure 3D**). *Clock* expression also showed tissue specific changes in that it no longer cycled in the gastrocnemius (**Table 3** **,** **Figure 3B**) but continued to do so in the liver (**Table 3**, **Figure 3E**) and the heart of hypertensive SHR (**Table 1**, **Figure S3E**).

**Table 3:** The circadian parameters of the core-clock genes *Bmal1*, *Clock*, and *Per2* in the gastrocnemius and liver of adult WKY and SHR calculated using JTK_CYCLE analysis.

Circadian Statistics Core Clock Genes				
Strain	Gene	JTK _CYCLE p value	Circadian JTK_CYCLE	JTK_CYCLE Amplitude
**Gastrocnemius**				
WKY Adult	*Bmal1*	7.40E-10	Yes	2.52
SHR Adult	*Bmal1*	1.20E-06	Yes	1.91
WKY Adult	*Clock*	1.68E-03	Yes	0.80
SHR Adult	*Clock*	7.82E-02	No	0.69
WKY Adult	*Per2*	1.24E-13	Yes	0.80
SHR Adult	*Per2*	3.92E-09	Yes	0.61
**Liver**				
WKY Adult	*Bmal1*	9.87E-12	Yes	1.95
SHR Adult	*Bmal1*	4.42E-09	Yes	0.75
WKY Adult	*Clock*	2.47E-05	Yes	0.55
SHR Adult	*Clock*	2.20E-09	Yes	0.74
WKY Adult	*Per2*	3.13E-09	Yes	0.40
SHR Adult	*Per2*	3.11E-08	Yes	0.29

**Figure 4:**
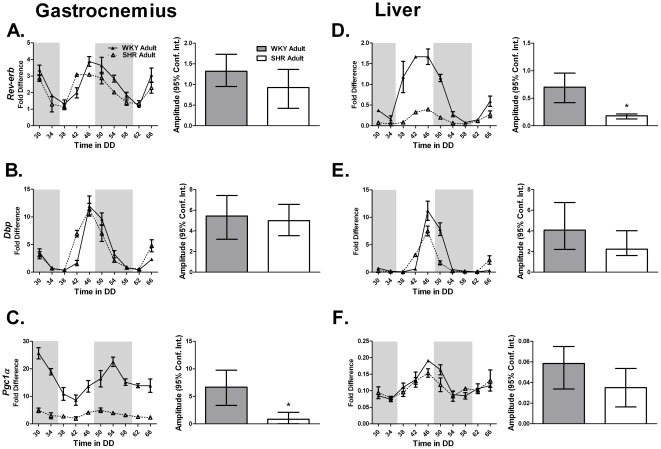
The expression of (A,D) *Rev-erbα*, (B) *Dbp*, and (C) *Pgc1α* in the gastrocnemius and liver of adult WKY and SHR was measured by quantitative PCR. Samples were collected every 4 hours for 40 hours. Collections were performed under red light. The dark and light bars on the graph represent extrapolated subjective day and night as defined by ZT according to the prior L∶D cycle before release into DD. A plot of amplitude with 95% confidence intervals as error bars is include next to each circadian gene expression plot.

*Rev-erbα* is a negative transcriptional regulator of *Bmal1* expression. Peak *Rev-erbα* expression was unchanged in skeletal muscle and heart (**Figure 4A** **,** **Table 4** **; Figure S4D, Table S2**). Paradoxically, *Rev-erbα* expression was damped coincident with decreased *Bmal1* expression in the liver of adult SHR (**Figure 4D** **)**. However, expression of *Pgc1α* was damped in the gastrocnemius but not the liver of the 22 week old SHR throughout the circadian time-course (**Figure 4C and F**).

**Table 4:** The circadian parameters of the core-clock gene *Rev-erb* and the clock-controlled genes *Dbp* and *Pgc1α* in the gastrocnemius and liver of adult WKY and SHR calculated using JTK_CYCLE analysis.

Circadian Statistics				
Strain	Gene	JTK _CYCLE p value	Circadian JTK_CYCLE	JTK_CYCLE Amplitude
**Gastrocnemius**				
WKY Adult	*Rev erb*	2.66E-09	Yes	1.32
SHR Adult	*Rev erb*	5.87E-06	Yes	0.93
WKY Adult	*Dbp*	5.22E-13	Yes	5.45
SHR Adult	*Dbp*	6.20E-09	Yes	5.00
WKY Adult	*Pgc1α*	5.87E-06	Yes	6.67
SHR Adult	*Pgc1α*	3.76E-05	Yes	0.86
**Liver**				
WKY Adult	*Rev erb*	2.00E-05	Yes	0.70
SHR Adult	*Rev erb*	4.78E-12	Yes	0.18
WKY Adult	*Dbp*	5.07E-10	Yes	4.07
SHR Adult	*Dbp*	1.20E-06	Yes	2.24
WKY Adult	*Pgc1α*	5.19E-06	Yes	0.06
SHR Adult	*Pgc1α*	2.66E-03	Yes	0.04

### Myod1 is dysregulated in the muscle of 22 week old SHR

**Figure 5:**
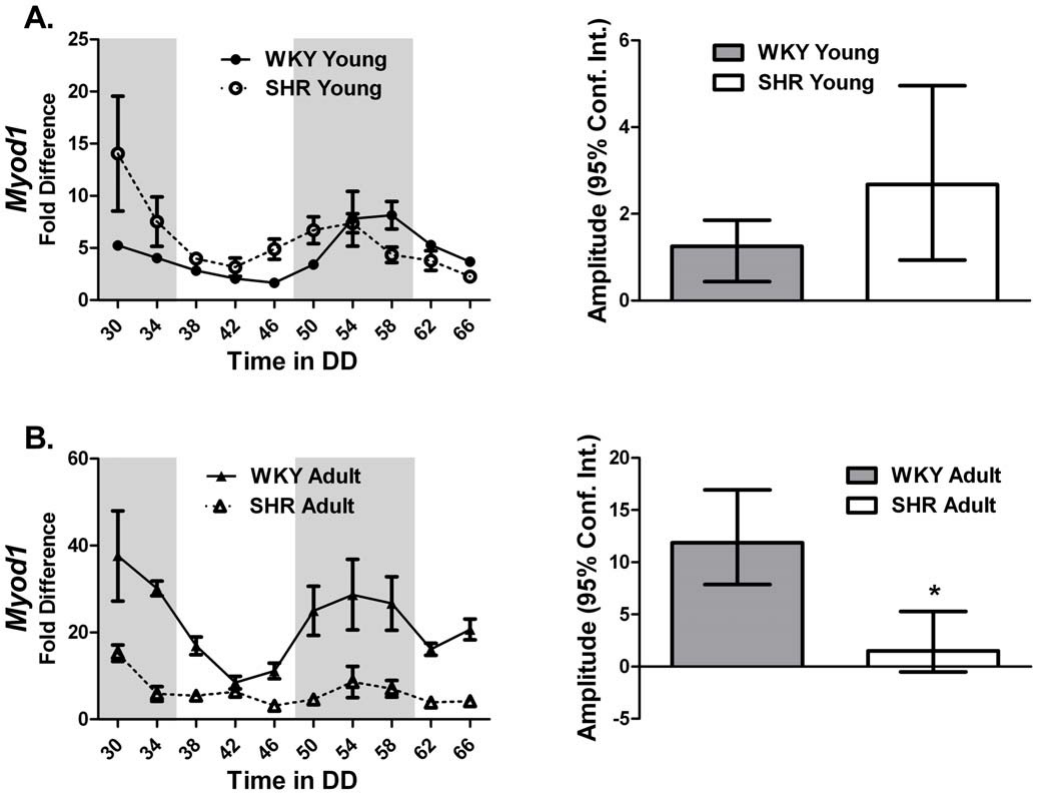
*Myod1* is a skeletal muscle specific clock-controlled gene. The expression of *Myod1* in the gastrocnemius of (A) young and (B) adult WKY and SHR was determined by quantitative PCR. Samples were collected every 4 hours for 40 hours. Collections were performed under red light. The dark and light bars on the graph represent extrapolated subjective day and night as defined by ZT according to the prior L∶D cycle before release into DD. Note that *Myod1* does not cycle in the gastrocnemius from adult SHR rats. A plot of amplitude with 95% confidence intervals as error bars is include next to each circadian gene expression plot.

*Myod1* is a well-described master regulator of the skeletal muscle transcriptional program [24]. JTK_CYCLE results confirm previously reported circadian cycling of *Myod1* (**Table 5**). Disruption of *Myod1* expression was severe in the adult 22 week old SHR animals. We found that expression of *Myod1* was no longer circadian and levels were damped throughout the circadian cycle (**Figure 5B**). Although little is known about the function of *Myod1* in adult skeletal muscle, the observation that *Myod1* oscillates is consistent with an expanded role in the daily maintenance of skeletal muscle tissue.

**Table 5:** The circadian parameters of the skeletal muscle specific clock-controlled gene *Myod1* in the gastrocnemius of young and adult WKY and SHR calculated using JTK_CYCLE analysis.

Circadian Statistics				
Strain	Gene	JTK _CYCLE p value	Circadian JTK_CYCLE	JTK_CYCLE Amplitude
**Gastrocnemius**				
WKY Young	*Myod1*	2.87E-04	Yes	1.25
SHR Young	*Myod1*	2.54E-06	Yes	2.68
WKY Adult	*Myod1*	8.79E-04	Yes	11.86
SHR Adult	*Myod1*	5.65E-02	No	1.52

**Figure 6:**
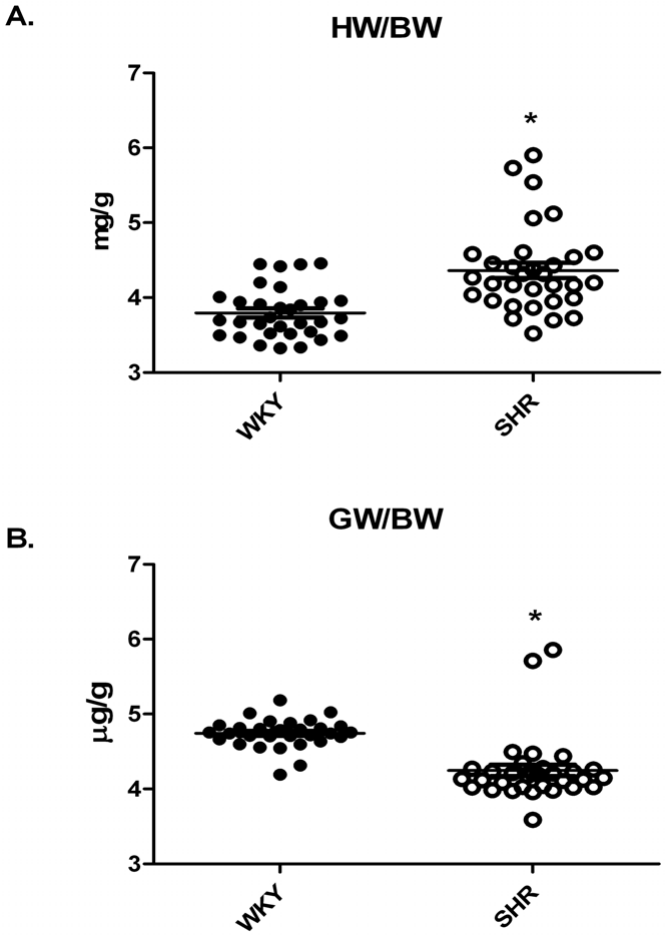
Heart weight normalized to body weight in the WKY and SHR demonstrating the presence of cardiac hypertrophy in the SHR (A). Gastrocnemius wet weight normalized to body weight measured from the hypertensive WKY and SHR demonstrating a loss of muscle mass in the SHR (**B**).

The presence of a hypertrophic heart was confirmed in that; heart weight normalized to body weight was significantly increased in the SHR (**Figure 6A**). Consistent with the observed loss of muscle mass in patients with cardiac hypertrophy/heart failure, the gastrocnemius muscle weight normalized to body weight of the SHR was significantly reduced compared to WKY (**Figure 6B**).

## Discussion

In this study, we examined a circadian time-course expression pattern of the molecular clock and clock-controlled genes in the gastrocnemius and liver in WKY and SHR rats at different disease stages to determine if polymorphisms upstream of the *Bmal1* gene in the SHR were associated with effects on the molecular clock either before or after disease onset. Overall, we found that there were differences in the expression of the molecular clock factors both pre- and post-disease onset but the patterns in different tissues varied across ages. The observation that molecular clock factor expression was altered across all peripheral tissues studied (skeletal muscle, liver and heart) prior to disease onset suggests that an underlying problem with the molecular clock may contribute to the both the hypertension and metabolic disease in the SHR rat. When looking at clock gene expression after disease onset, we found more pronounced effects in skeletal muscle with a loss of *Clock* cycling and a dampening of the clock-controlled genes *Pgc1α* and *Myod1*. The damped expression of *Myod1* and *Pgc1α* in the hypertensive and insulin resistant SHR was associated with a significant loss of muscle mass. Examination of single time point gene expression in tissues from heart failure SHR rats revealed significant down-regulation of several molecular clock genes in skeletal muscle as well as genes responsible for daily muscle maintenance and function. In summary, these findings confirm that molecular clock expression in the SHR is disrupted prior to the onset of cardiovascular and metabolic disease. The continued disruption in clock and clock-controlled genes in the skeletal muscle following disease onset suggests that the expression and/or stability of the molecular clock may contribute to the pathophysiology phenotype seen in this model.

### Core-clock disruption precedes the development of hypertension

Our studies in the WKY and SHR demonstrate a pattern of core-clock disruption beginning in the pre-hypertensive SHR. Hypertension and metabolic disease are systemic diseases involving many organs beyond vasculature and heart. Subtle disruption of the molecular clock through changing peak amplitude (*Per2*, *Rev-erbα*) may be characteristic of an unstable clock mechanism in these tissues thus, making the tissues/organs more prone to disease triggers and increasing the probability of disease onset. Since the clock mechanism is not totally lost upon disease onset we favor a model in which there is a delicate time-dependent balance of expression of core-clock genes necessary to diminish the potential for developing pathology. An example of subtle changes in core-clock expression patterns contributing to pathology occurs in human breast cancer. Dysregulation or loss of PER1 and/or PER2 has been shown in human breast cancer tumor cells when compared with normal cells from adjacent tissue [25]. Lack of PER synchrony was even observed in the expression patterns of PER in separate cancer cell populations of the same cancer tissue. In addition, many clinical studies have associated circadian regulation/synchrony with cancer [25]–[27]. Studies in liver and adipose tissue of ob/ob mice have also shown that dysregulation of *Per1*, *Per2* and *Dbp* occurs prior to the development of metabolic abnormalities suggesting a causal link [28]. These data under-score the need for future studies focusing on targeting the molecular clock to stabilize phase and amplitude and coordinate the circadian system.

### Hypertension and insulin resistance is associated with pronounced changes of the core-clock in the gastrocnemius muscle

As the SHR progressed from pre-hypertensive to hypertensive and insulin resistant (22 Weeks, adult), the disruption of the molecular clock was more pronounced in the gastrocnemius compared to the liver. The tissue specificity of the clock disruption in skeletal muscle suggests 1) there is likely a gene – environment interaction leading to tissue specific disruption of the molecular clock; 2) clock gene disruption in skeletal muscle is associated with insulin resistance and 3) clock disruption of skeletal muscle likely contributes significantly to the whole body insulin resistance in this model. The SHR strain has been used extensively as a genetic model of essential hypertension. Skeletal muscle is a primary site of insulin resistance in essential hypertension [29], [30] It is interesting to note that SHR exhibit several skeletal muscle abnormalities and dysfunctions when compared to WKY counterparts including decreased fatigue resistance [31], insulin resistance [32], development of less contractile force [33], increased interstitial norepinephrine levels [34], altered sodium pump number and activity [35], elevated intracellular free calcium [36], fiber type transformation [37], and decreased capillary density [38]. Major alterations in skeletal muscle ultrastructure and biochemical properties have also been demonstrated in patients with chronic heart failure including fiber atrophy [17], transformation of fast-twitch type I to slow-twitch type II fibers [18], [39], decrease in oxidative enzyme capacity [19] and abnormal mitochondrial structure [20]–[22]. Recent studies in two clock-compromised mouse strains identified a common skeletal muscle pathology that was characterized by diminished force capacity and reduced mitochondrial content and function [5] both of which have been observed in SHR. Loss of *Clock* cycling in the hypertensive SHR provide insights that that modification of core-clock components may contribute to the functional and metabolic phenotype observed in SHR skeletal muscle. Metabolic dysfunction may also manifest through decreased expression of *Rev-erb* in the SHR gastrocnemius. The orphan nuclear receptors *Rev-erb* and *Rora* (RAR-related orphan receptor) link the feedback loops of the clock by repressing and activating *Bmal1* gene transcription, respectively [40]–[43]. *Rora* and *Rev-erb* have also been shown to regulate lipid homeostasis in skeletal muscle [44], [45]. Changes in the expression of these genes could have a broad impact beyond circadian regulation, in that skeletal muscle relies heavily on fatty acids as a fuel source.

### Circadian expression of Myod1 and Pgc1α is disrupted in the hypertensive gastrocnemius

Recent work from our lab has demonstrated that expression of *Myod1* oscillates in a circadian pattern in skeletal muscle [10] and is a direct clock-controlled gene [5]. Cycling of *Myod1*, a direct target of the core-clock genes *Bmal1* and *Clock* in skeletal muscle, ceased in the gastrocnemius muscle of the hypertensive and insulin resistant SHR. While speculative, the loss in gastrocnemius muscle weight in the hypertensive SHR may be connected to the disruption in *Myod1* expression [46]. Another gene, *Pgc1α*, known to play a critical role in regulating the expression of metabolic and mitochondrial genes in skeletal muscle was damped in the gastrocnemius of the hypertensive rats throughout the circadian collection time-course. Data from Patti and colleagues is highly suggestive of a potential link between PGC1 expression and insulin resistance in diabetic and non-diabetic individuals with a family history of diabetes [47]. A reduction of the transcriptional co-activator *Pgc1α* in the gastrocnemius of the hypertensive SHR is indicative of a role for the molecular clock in the insulin resistance observed in these hypertensive animals. This work is in agreement with previous work from Andrews et al., (2010) demonstrating a down-regulation of both *Pgc1α* and *Pgc1ß* in mice with disruption of the core-clock genes *Clock^Δ19^* or *Bmal1^−^* ^/−^. These mice exhibit altered mitochondrial structure and function.

### Multiple core-clock components are down regulated in skeletal muscle of heart failure SHR

Our data revealed a pattern of down-regulation of several core-clock genes, including *Clock* and *Bmal1*, and non-circadian skeletal muscle genes, such as *Mef2* and *Ttn*, responsible for daily muscle maintenance in the gastrocnemius of the SHR during overt heart failure. Work from this lab recently reported that muscle from *Bmal1^−/−^* and *Clock^Δ19^* mice exhibit disrupted myofilament architecture and exhibit reduced normalized maximal force [5] demonstrating a role for the molecular clock in maintaining structure and function at the cellular level in skeletal muscle. However, the skeletal muscle specific circadian gene *Myod1* was unchanged in the heart failure animals (**Figure S1A**). This finding may more reflect a limitation of the experimental design rather than the biology. Given the high mortality of the heart failure animals, it was only possible to collect at a single time point. Tissue for heart failure experiments were collected during the daylight hours and this lack of change may be more a result of the time of day of collection. As seen in **Figure 5B**, *Myod1* expression in the gastrocnemius from the WKY and hypertensive SHR shows that *Myod1* expression levels were not different during the daylight hours. Our observation that *Myod1* target genes *Mck* and *Ttn*, were down-regulated is suggestive that the loss of oscillation of *Myod1* in the SHR is maintained in the heart failure rats.

Patterns of dysregulation in the molecular clock including diminished amplitude, phase shifts, and period changes have been observed in many disease states like diabetes, cardiovascular disease and cancer [4], [48]–[52]. Dyssynchrony can be the result of genetic alterations or changes in environmental cues. As a whole, our data demonstrate a pattern of molecular clock dysregulation that begins prior to the development of hypertension and insulin resistance and may be linked to the genetic background of the SHR progressing independent of the development of hypertension or worsening because of the development of hypertension. In association with previous studies, these data are highly suggestive of a role for the molecular clock in skeletal muscle pathology associated with hypertension/hypertrophy in SHR. Whether these genetic variables are identical or overlap with those in hypertensive patients is unknown. Future studies will need to examine other models of hypertension in order to better understand the mechanism behind hypertension, heart failure and circadian gene regulation in skeletal muscle.

## Methods

### Animals

All animal procedures were conducted in accordance with institutional guidelines for the care and use of laboratory animals as approved by the University of Kentucky Institutional Animal Care and Use Committees. This work was approved by the Institutional Animal Care and Use Committee (IACUC) at the University of Kentucky, protocol number 00890M2005. WKY and SHR rats were obtained from Harlan Laboratories. Rats were housed in a temperature- and humidity-controlled room maintained on a 12 h light–12 h dark cycle with food and water ad libitum.

### Tissue Collections (80 wks old)

Due to the high mortality of the heart failure rats (80 wks old) tissue samples were collected at a single time point, Tissue samples from old rats (80 wks old) were collected at the same time of day, during 8AM and 9AM. This corresponds to time between ZT1 and ZT2.

### Circadian Collections

All rats were maintained in the 12 h L/D cycles, then released into DD. Starting 30 h after entry into DD skeletal muscle, heart and liver from three WKY and three SHR rats were collected every 4 h for 40 h under dim red light (<5 lux). The muscles, liver and heart were removed from each rat and frozen in liquid nitrogen.

### RNA isolation

Total RNA was prepared from frozen tissue samples using TRIzol (Invitrogen) according to the manufacturer's directions. RNA samples were treated with TURBO DNase (Ambion, Austin, TX) to remove genomic DNA contamination. Isolated RNA was quantified by spectrophotometry (λ = 260 nm). First-strand cDNA synthesis from total RNA was performed with a mixture of oligo(dT) primer and random hexamers using SuperScript III First-Strand Synthesis SuperMix (Invitrogen). All isolated RNA and cDNA samples were stored at −80°C until further analysis. Real-time quantitative PCR using TaqMan (Applied Biosystems) assays was used to examine the gene expression of several core-clock, clock-controlled, and skeletal muscle specific genes. The gene expression assays were as follows: *Bmal1* Rn00562847_m1*; *Per2* Rn01427704_m1*; *Clock* Rn00573120_m1*; *Rora* Rn01173769_m1*; *Rev- erb* Rn00595671_m1*; *Myod* Rn00591291_m1*; *Pgc1α* Rn00580241_m1*; *Rpl26* Rn02127510_s1*; *Mck* Rn01644605_m1*; *Dbp* Rn00497539_m1*. RPL26 was used as an internal calibration control [5], [10], [11], [53]. The ΔΔCT method was used for the quantification of real-time PCR data in the circadian collections. Gene expression in each sample was shown as the relative value compared to the WKY soleus sample No. 1 from DD 30 hours group. This enabled us to compare not only inter-species differences but also inter-organ differences in the expression pattern/amplitude of each gene.

#### Statistics

A new version of JTK_CYCLE was used to identify and characterize cycling variables in our datasets [23]. JTK_CYCLE analyses were performed using the R statistical package, version 2.12.1 to look specifically for 24 hour rhythms. Briefly, the original version of JTK_CYCLE estimated the amplitude of the most probable period/lag combination by calculating the median sign-adjusted deviation from the median over the first complete cycle. The new version-2 of JTK_CYCLE provides a more precise estimate of amplitude by replacing the median with the “pseudo median” (Hodges-Lehmann estimator) and additionally reports the associated confidence interval calculated by the Wilcox test function in the standard R stats package. A clear explanation of the mathematical basis for these new features is available online [54]. Differences in heart weight/body weight ratios and gastrocnemius wet weight/body weight were calculated with Graphpad Prism software using a two-tailed student t test.

## Supporting Information

Figure S1 ***Bmal1*** **,** ***Clock*** **,** ***Per2*** **and** ***Rora*** **are core-clock genes.** Expression of (A) *Bmal1*, (B) *Clock*, (C) *Per2*, and (D) *Rora* from the gastrocnemius of WKY and SHR in heart failure (80 weeks) was determined by quantitative PCR. (*p<0.05).
(TIF)

Figure S2 **The expression of the skeletal muscle genes (A)** ***Myod1*** **, (B)** ***Mck*** **, (C)** ***Mef2c*** **, and (D)** ***Ttn*** from the gastrocnemius of WKY and SHR in heart failure (80 weeks) was determined by quantitative PCR. (*p<0.05).
(TIF)

Figure S3 **The expression of (A,D)** ***Bmal1*** **, (B,E)** ***Clock*** **, and (C,F)** ***Per2*** **from the heart of young and adult WKY and SHR was determined by quantitative PCR.** Samples were collected every 4 hours for 40 hours. Collections were performed under total red light. The dark and light bars on the graph represent extrapolated subjective day and night as defined by ZT according to the prior L∶D cycle before release into DD. A plot of amplitude with 95% confidence intervals as error bars is include next to each circadian gene expression plot.
(TIF)

Figure S4 ***Rev-erbα*** **is a core-clock gene and** ***Dbp*** **, and** ***Pgc1α*** **are clock-controlled genes.** The expression of (A,D) *Rev-erb*, (B,E) *Dbp*, and (C,F) *Pgc1α* in the heart of young and adult WKY and SHR rats was measured by quantitative PCR. Samples were collected every 4 hours for 40 hours. Collections were performed under red light. The dark and light bars on the graph represent extrapolated subjective day and night as defined by ZT according to the prior L∶D cycle before release into DD. A plot of amplitude with 95% confidence intervals as error bars is include next to each circadian gene expression plot.
(TIF)

Table S1 **The circadian parameters of the core-clock genes** ***Bmal1*** **,** ***Clock*** **, and** ***Per2*** **in the heart of young and adult WKY and SHR calculated using JTK_CYCLE analysis.**
(TIF)

Table S2 **The circadian parameters of the clock-controlled genes** ***Rev-erb*** **,** ***Dbp*** **, and** ***Pgc1α*** **in the heart of young and adult WKY and SHR calculated using JTK_CYCLE analysis.**
(TIF)
