# Supplementary figures and images for: Age-Associated Disruption of Molecular Clock Expression in Skeletal Muscle of the Spontaneously Hypertensive Rat
Source: PLoS One. 2011 Nov 4;6(11):e27168. doi: 10.1371/journal.pone.0027168 (PMC3208587; doi:10.1371/journal.pone.0027168)

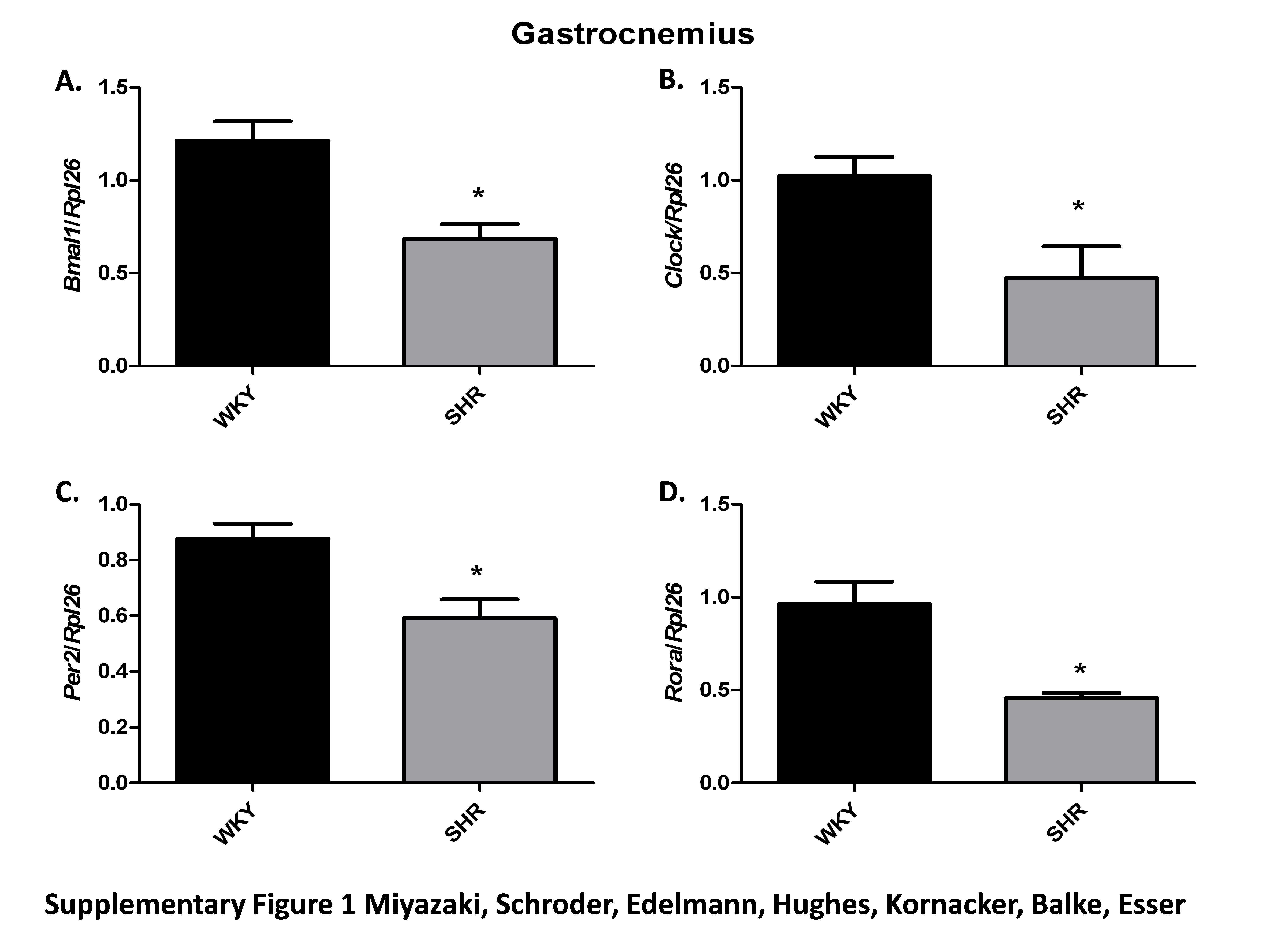

Supplement: Figure S1 — Bmal1 , Clock , Per2 and Rora are core-clock genes. Expression of (A) Bmal1, (B) Clock, (C) Per2, and (D) Rora from the gastrocnemius of WKY and SHR in heart failure (80 weeks) was determined by quantitative PCR. (*p<0.05). (TIF) [file pone.0027168.s001.tif]

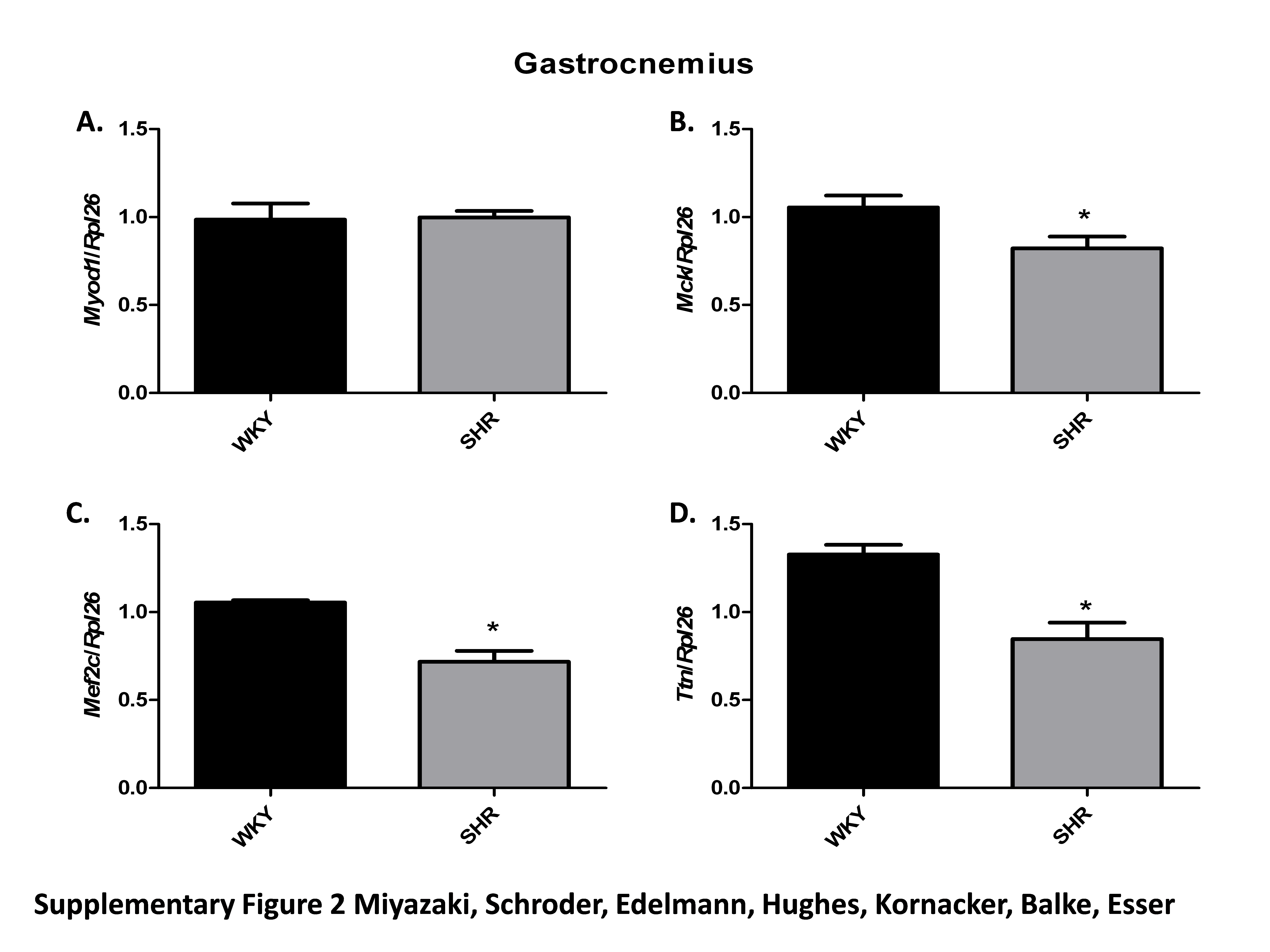

Supplement: Figure S2 — The expression of the skeletal muscle genes (A) Myod1 , (B) Mck , (C) Mef2c , and (D) Ttn from the gastrocnemius of WKY and SHR in heart failure (80 weeks) was determined by quantitative PCR. (*p<0.05). (TIF) [file pone.0027168.s002.tif]

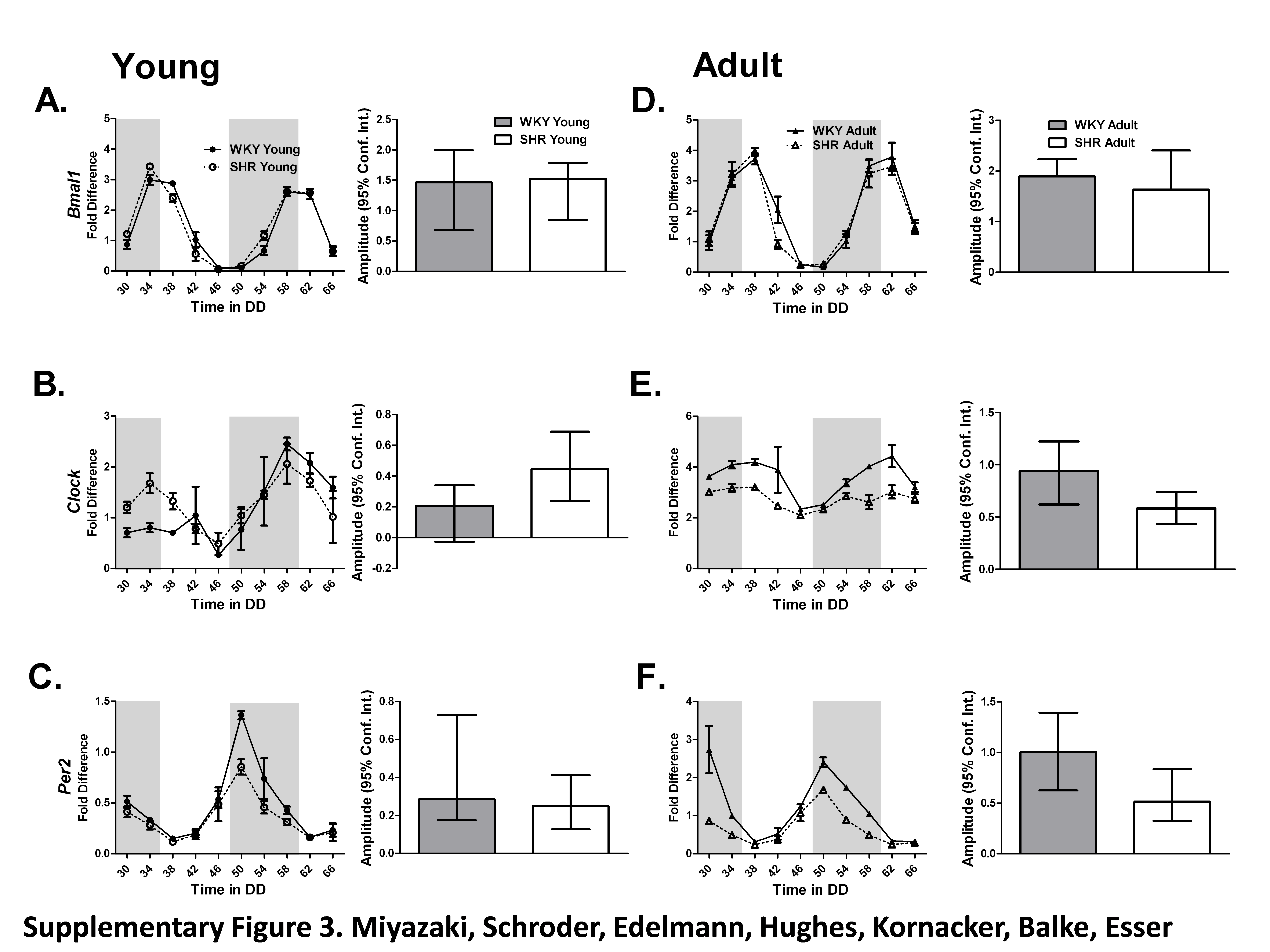

Supplement: Figure S3 — The expression of (A,D) Bmal1 , (B,E) Clock , and (C,F) Per2 from the heart of young and adult WKY and SHR was determined by quantitative PCR. Samples were collected every 4 hours for 40 hours. Collections were performed under total red light. The dark and light bars on the graph represent extrapolated subjective day and night as defined by ZT according to the prior L∶D cycle before release into DD. A plot of amplitude with 95% confidence intervals as error bars is include next to each circadian gene expression plot. (TIF) [file pone.0027168.s003.tif]

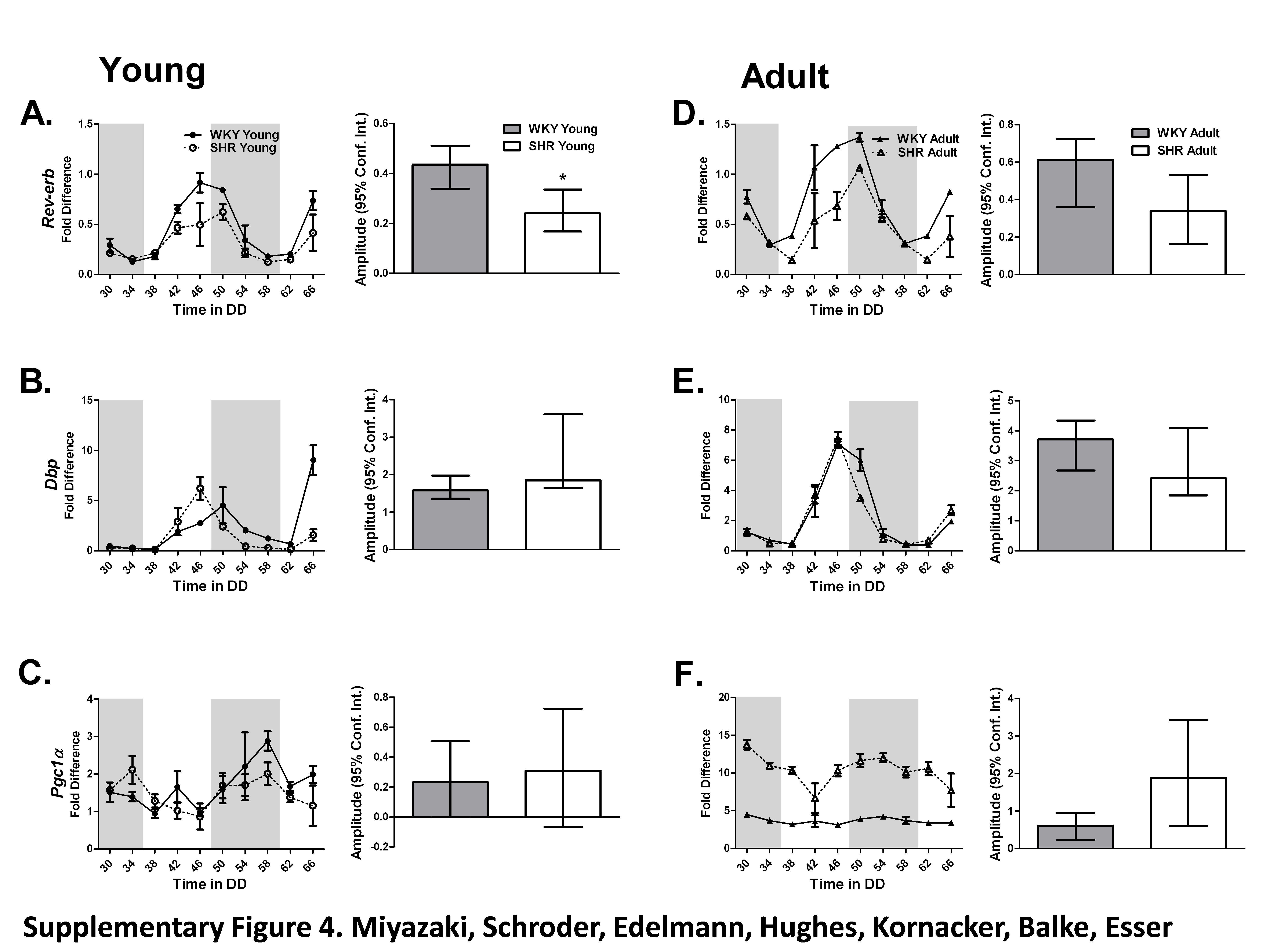

Supplement: Figure S4 — Rev-erbα is a core-clock gene and Dbp , and Pgc1α are clock-controlled genes. The expression of (A,D) Rev-erb, (B,E) Dbp, and (C,F) Pgc1α in the heart of young and adult WKY and SHR rats was measured by quantitative PCR. Samples were collected every 4 hours for 40 hours. Collections were performed under red light. The dark and light bars on the graph represent extrapolated subjective day and night as defined by ZT according to the prior L∶D cycle before release into DD. A plot of amplitude with 95% confidence intervals as error bars is include next to each circadian gene expression plot. (TIF) [file pone.0027168.s004.tif]

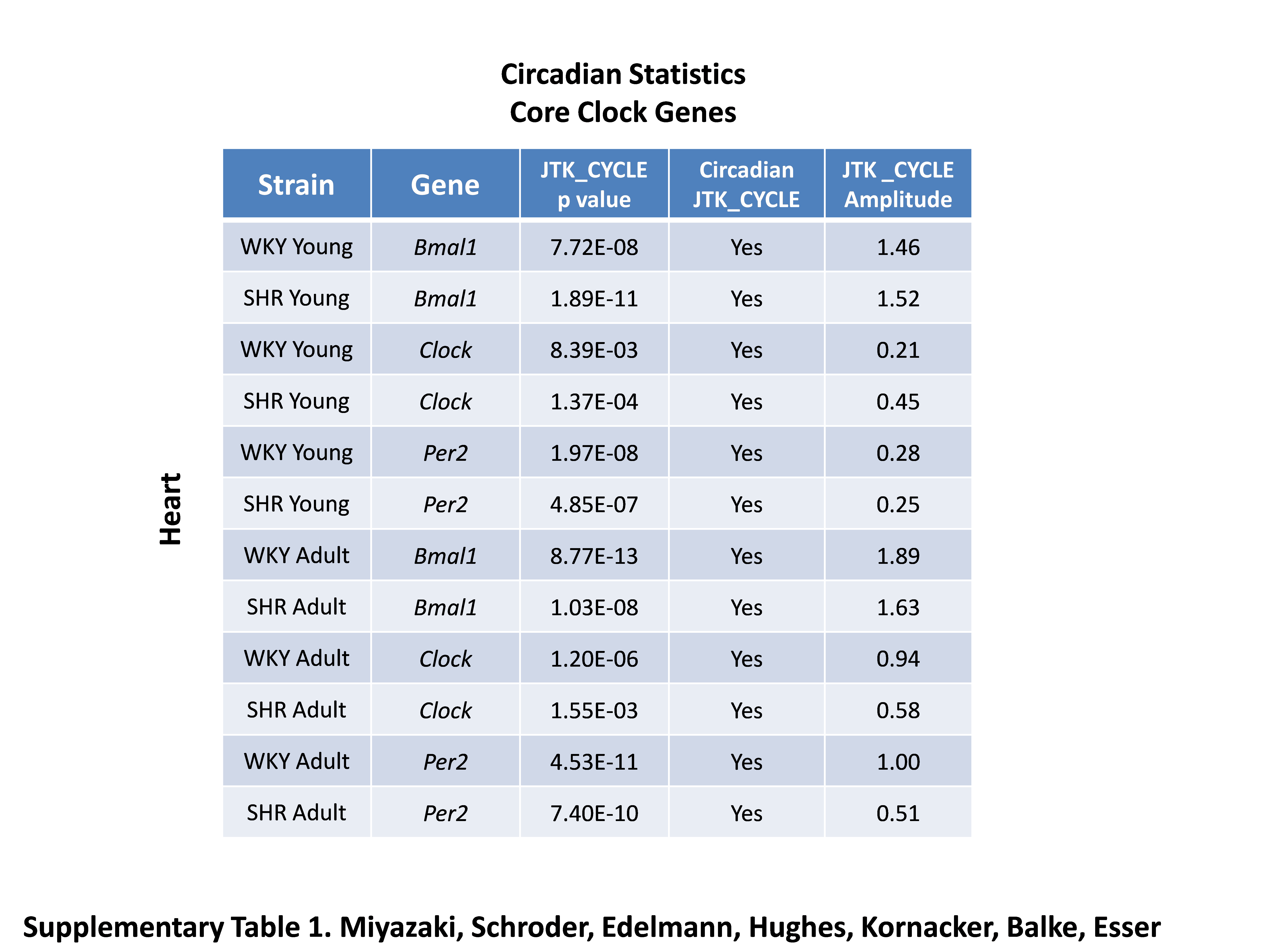

Supplement: Table S1 — The circadian parameters of the core-clock genes Bmal1 , Clock , and Per2 in the heart of young and adult WKY and SHR calculated using JTK_CYCLE analysis. (TIF) [file pone.0027168.s005.tif]

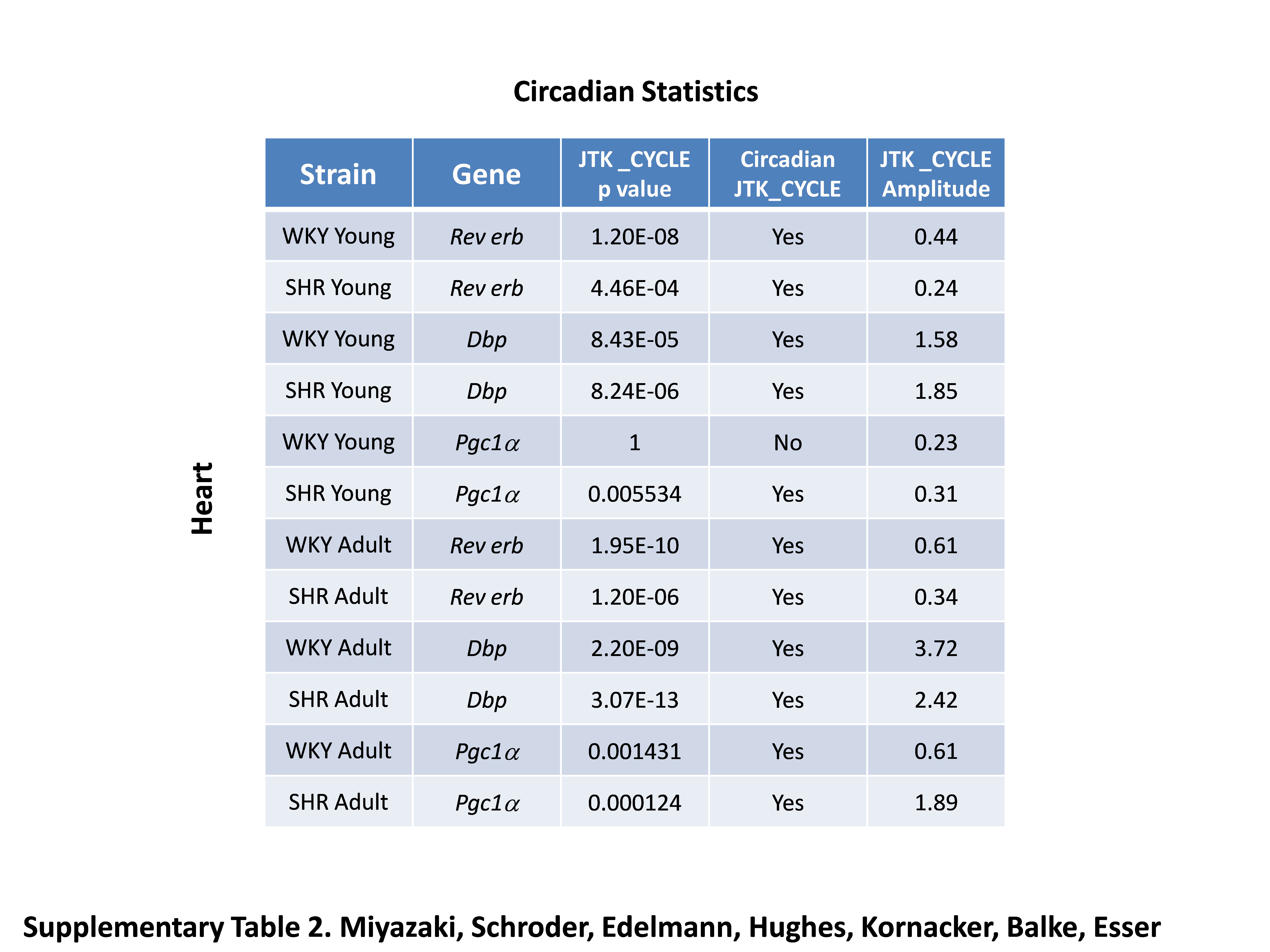

Supplement: Table S2 — The circadian parameters of the clock-controlled genes Rev-erb , Dbp , and Pgc1α in the heart of young and adult WKY and SHR calculated using JTK_CYCLE analysis. (TIF) [file pone.0027168.s006.tif]
